# Klaus Winter – the indefatigable CAM experimentalist

**DOI:** 10.1093/aob/mcad028

**Published:** 2023-04-03

**Authors:** Joseph A M Holtum

**Affiliations:** College of Science and Engineering, James Cook University, Townsville, Queensland 4811, Australia

**Keywords:** Biography, crassulacean acid metabolism (CAM), facultative CAM, inducible CAM, Klaus Winter, *Mesembryanthemum crystallinum*, Smithsonian Tropical Research Institute (STRI)

## Abstract

**Background:**

In January 1972, Klaus Winter submitted his first paper on crassulacean acid metabolism (CAM) whilst still an undergraduate student in Darmstadt. During the subsequent half-century, he passed his Staatsexamensarbeit, obtained his Dr. rer. nat. *summa cum laude* and Dr. rer. nat. habil., won a Heinz Maier-Leibnitz Prize and a Heisenberg Fellowship, and has occupied positions in Germany, Australia, the USA and Panama. Now a doyen in CAM circles, and a Senior Staff Scientist at the Smithsonian Tropical Research Institute (STRI), he has published over 300 articles, of which about 44 % are about CAM.

**Scope:**

I document Winter’s career, attempting to place his CAM-related scientific output and evolution in the context of factors that have influenced him as he and his science progressed from the 1970s to the 2020s.

## THE STUDENT YEARS

Klaus Winter was introduced to the international scientific community, and the crassulacean acid metabolism (CAM) community in particular, with his first publication, now a CAM classic with over 230 citations. ‘NaCl-induzierter Crassulaceensäurestoffwechsel bei *Mesembryanthemum crystallinum*’ (Winter and von Willert, 1972) was submitted to *Zeitschrift für Pflanzenphysiologie* on 11 January 1972, when Klaus was still an undergraduate student at the Technical University of Darmstadt (Winter, 1972). An account of his discovery of what is now considered the archetypal facultative-CAM species is given in Winter (2023).

Examination of chapter topics and methods of his Staatsexamensarbeit and his subsequent Dr. rer. nat. dissertation, which began in early 1973, illustrates well the advantages afforded early-career ecophysiologists investigating CAM. His analyses of the interactions between salinity and the photosynthetic pathway in halophytes, particularly CAM induction in *M. crystallinum*, involved studying under a range of conditions, plants growing in soil or hydroponics in pots, controlled climate cabinets, glass-houses, and in the field in Israel. He performed a wide range of techniques, including gas exchange, enzyme assays, ^14^C-labelling, leaf anatomy, determination of malate, Na^+^, K^+^ and Cl^−^, water relation parameters and microclimate measurements. This corpus of techniques, and mastering the theoretical and practical understanding required to use them well, has underpinned many of Klaus’ subsequent publications in the laboratory and field ecophysiology of both CAM and non-CAM plants.

A capacity for prolific publication was quickly evident. As a doctoral student, Klaus co-authored a *Nature* paper on CAM in 1973 (Osmond *et al.*, 1973), the first of many collaborations with antipodean scientists. He subsequently submitted five manuscripts, four of which were single-authored, on salt-induced CAM (Winter, 1973*a*, *b*, *c*, *d*; Winter *et al.*, 1974), and a further four papers in 1974 (Winter, 1974*a*, *b*, *c*, *d*). He submitted his Dr. rer. nat. dissertation in May, 1975 (Winter, 1975). It was graded *summa cum laude*, of course!

## THE FIRST POSTDOC

In 1976 and 1977, Klaus remained in Ulrich Lüttge’s laboratory in Darmstadt under the auspices of a Deutsche Forschungsgemeinschaft (DFG) Wissenschaftlicher Mitarbeiter position. Two themes dominated his research output during this period – pursuing his biochemical and physiological mechanistic investigations of CAM halophytes and a return to the field in Israel for seasonal studies of CAM

**Fig. 1.**
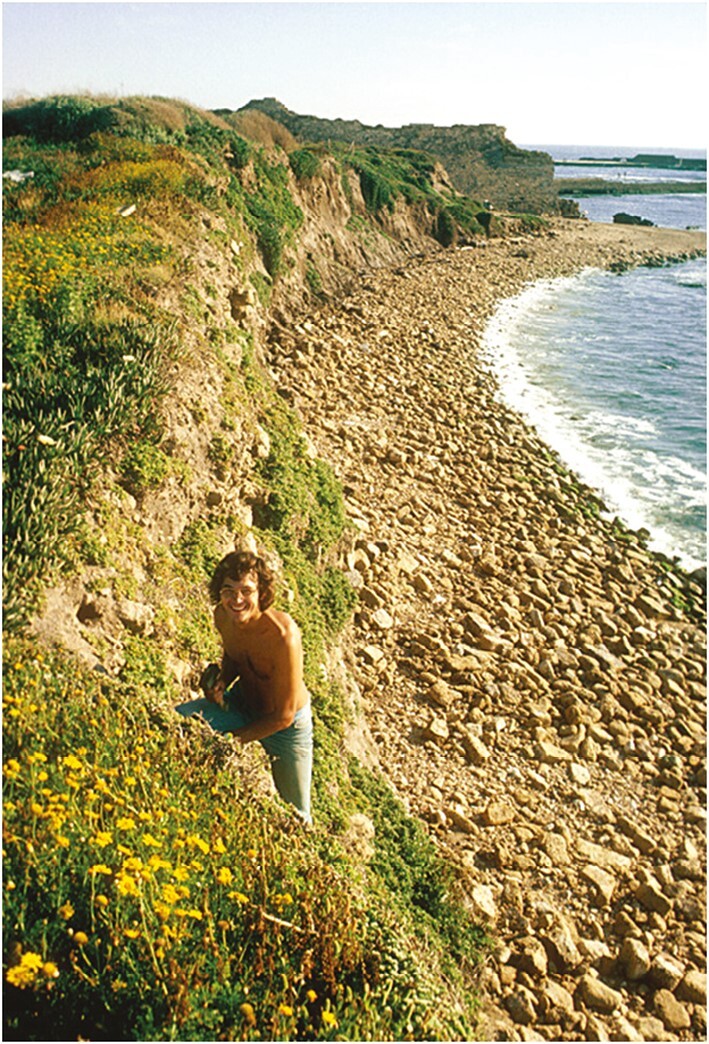
Klaus Winter perched among *Mesembryanthemum* plants growing on a coastal cliff near Caesarea in Israel, 1977. Photo: K. Winter.

Klaus’ antipodean connections with plants in their natural environments (Fig. 1; Winter *et al.*, 1978) continued in Darmstadt when he met and collaborated with Hank Greenway, a gifted critical- and lateral-thinking Australian-based plant physiologist who was visiting under the auspices of a DFG Richard-Merton Guest Professorship (Greenway *et al.*, 1978; Winter and Greenway, 1978). Hank’s unalloyed enjoyment of the thrill of science and his preparedness to share and debate ideas particularly impressed Klaus, as indeed it affected many other colleagues and students throughout Hank’s career (Atwell *et al.*, 2021).

Klaus published the first of what was to become a career-long intermittent series of carbon isotope and titratable acidity studies of succulent and non-succulent plants in the wild (Winter *et al.*, 1976*a*, *b*; Winter and Troughton, 1978*a*, *b*; Holtum *et al.*, 2016; Pachon *et al.*, 2022). Among these was another CAM classic that traced the seasonal change of isotopic composition and nocturnal malic acid increase in *M. crystallinum* switching from C_3_ to CAM photosynthesis in its natural environment (Winter *et al.*, 1978). He also published his first non-CAM publication, the first report of C_4_ photosynthesis in the Polygonaceae (Winter *et al.*, 1977), a discovery initially made during his doctoral research. Included in the latter paper was a reference to Volkens (1887), ‘Die Flora der aegyptisch-arabischen Wüste’, in which Kranz anatomy was illustrated around the time that Haberlandt (1884) coined the now-popular linguistically mixed term ‘Kranz anatomy’.

## A SOJOURN DOWN UNDER

In 1978, Klaus accepted a postdoctoral fellowship in Canberra with Barry Osmond in the Department of Environmental Biology, Research School of Biological Sciences (RSBS) at the Australian National University (ANU). A progressive funding model, by which RSBS was funded directly by a block grant from the Australian federal government, coupled with astute hiring and management by the school’s director, Sir Bob Robertson, and the head of department, Ralph Slatyer, had resulted in RSBS being at the cutting edge of multiple important expanding areas of international photosynthetic and ecophysiological research, all of which Klaus could tap into and from which he could gain experience. George Lorimer, John Andrews and Murray Badger were unravelling the chemistry of the oxygenase reaction of Rubisco and establishing the biochemistry of photorespiration. Ralph Slatyer, Ian Cowan and Grahame Farquhar, with students Suan Chin Wong, Marilyn Ball, Steve Powles and Susanne von Caemmerer, were using sophisticated gas-exchange systems to explore the biophysical and mechanistic bases of plant water movement and its control, and extrapolating their observations to explore components of plant isotope fractionation, photodamage and photoprotection. Barry Osmond’s group was at the forefront of research into CAM and photosynthetic nitrogen metabolism (Sutton, 1975; Osmond, 1978; Woo and Osmond, 1982). The mass spectrometry unit, managed by Roger Summons and subsequently Zarko Roksandic, was on top of emerging new techniques of stable isotope analyses. Les Watson and PhD student Paul Hattersley were cataloguing Australia’s C_4_ variety (Hattersley and Watson, 1976) while across campus, at CSIRO Plant Industry, Hal Hatch with postdoc Jim Burnell and students Bob Furbank and Stuart Boag were unlocking the biochemistry of the C_4_ pathway.

**Fig. 2.**
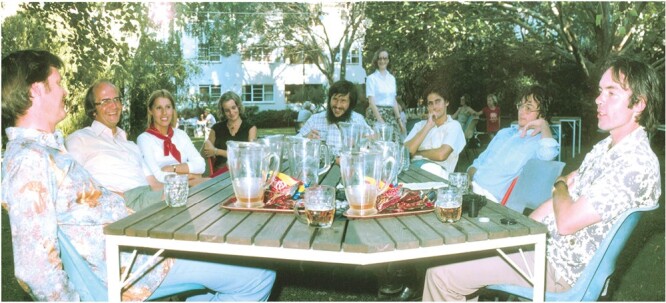
Enjoying Nobby’s Nuts and the sunshine in the Fellows’ Garden of University House, ANU, 1978. From left to right: Olle Björkman, Otto Lange, Erika Winter, Rosemary Dunn, Joe Holtum, Jennifer Holmgren, Steve Powles, Klaus Winter and Grahame Farquhar. Photo: S. C. Wong.

The calibre of plant scientists in Canberra, and the freedom of academic pursuit there, fostered scientific exchange and attracted overseas visitors (Fig. 2; Osmond, 1995, 2014). In 1978–79 alone, the who’s who of eminent overseas visitors to Environmental Biology included Andy Benson, Joe Berry, Olle Björkman, Dave Canvin, Heinrich Fock, Heinrich Krause, Otto Lange, Malcolm Nobbs, Marion O’Leary, John Raven and Irwin Ting. It was in Canberra that Klaus, rather stylish in dress and quiet in manner, first met and collaborated with Joe Holtum, a hirsute, scruffy, rugby-playing PhD student supervised by Barry Osmond (Fig. 2). By 2022 the unlikely pair had co-authored 39 manuscripts on CAM.

Klaus flourished in this heady academic environment, learning methods, absorbing rapidly evolving ecophysiological/physiological theory, and making important connections. He continued his focus upon unravelling the physiology and biochemistry of *M. crystallinum*, particularly phospho*enol*pyruvate carboxylase (PEPC), that he began with Greenway in Darmstadt. ‘Working the night shift’ (Black and Osmond, 2003) for several months with Beethoven cranked up to 11, Klaus produced more CAM biochemistry classics with his demonstration of the changes in the *K*_*m*PEP_ and *K*_*i*malate_ of PEPC from CAM *M. crystallinum* during day–night cycles (Winter, 1981*a*, 1982). This research demonstrated that PEPC is unlikely to be functional during deacidification in the light as it is maintained in a state that is highly sensitive to malate inhibition. The experiments were intense: in order to be sure that the form of the enzyme being studied was that in tissue, extraction, desalting and assay of PEPC within 2 minutes was required. George Lorimer, a biochemist, was not impressed with Klaus blasting plant extracts under non-equilibrium conditions through Sepahadex-G25 under pressure but, as demonstrated in an associated methods paper (Winter, 1980), Klaus’ system worked.

With John Pate, Klaus continued his field isotopic CAM exploration studies with the first survey of semi-arid areas of Western Australia (Winter *et al.*, 1981*b*), showing CAM-type isotopic values in *Calandrinia*, an observation that has been pursued in recent years (Winter and Holtum, 2011; Hancock *et al.*, 2018, 2019).

**Fig. 3.**
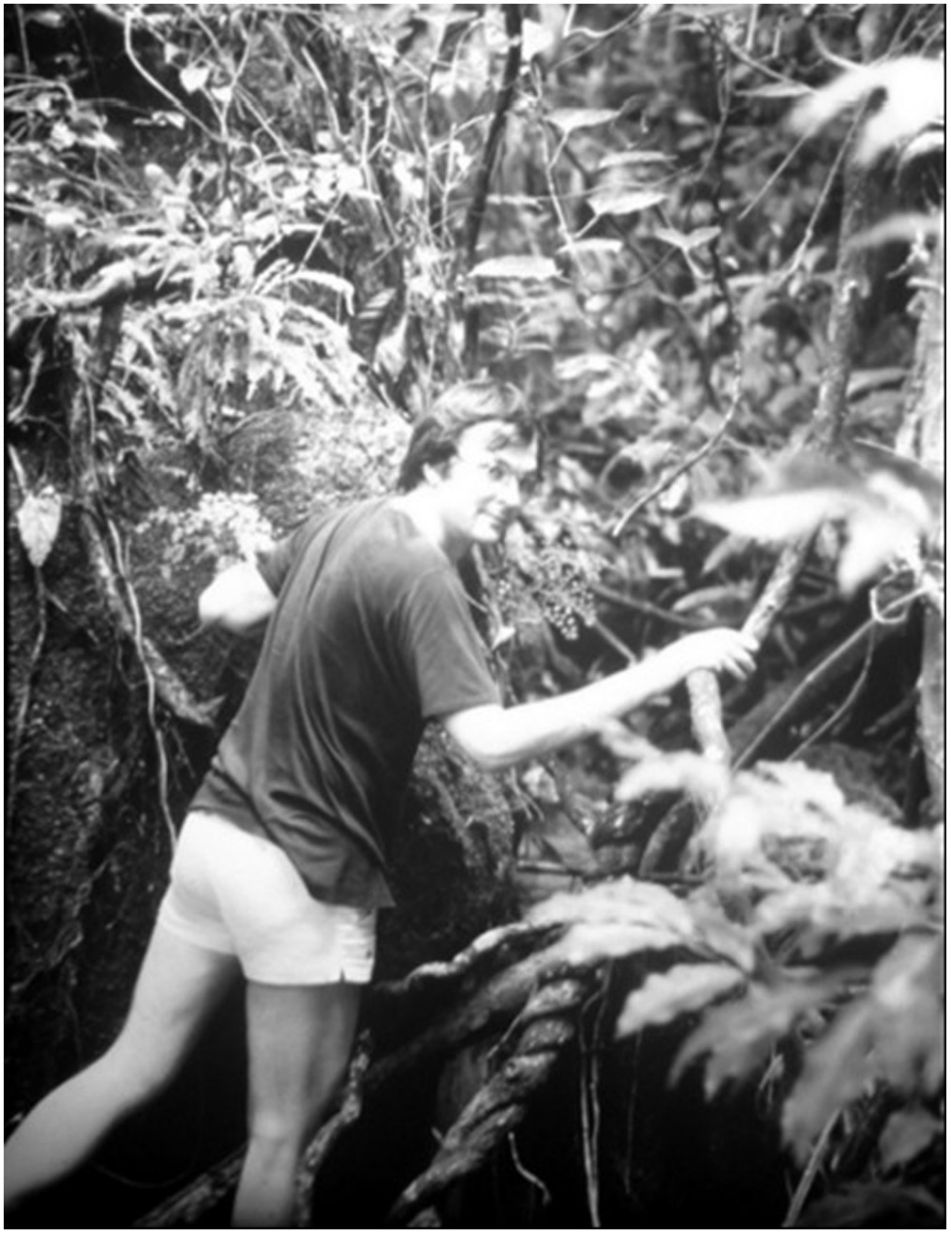
Klaus Winter and epiphytes in an eastern Australian rainforest, 1978 or 1979. Photo: C. B. Osmond and C. Büchen-Osmond.

He expanded his isotopic surveys to include tropical and subtropical wet forests, for which disparate evidence of CAM in epiphytes was emerging (Nuernbergk, 1961; Coutinho, 1963; McWilliams, 1970; Medina and Troughton, 1974; Neales and Hew, 1975; Wong and Hew, 1976). In the first survey of epiphytes in the rainforests of eastern mainland Australia Klaus demonstrated CAM in orchids and *Pyrrosia* ferns and reported CAM for the first time in the rubiaceous ant-plants *Mymecodia* and *Hydnophytum* (Fig. 3; Winter *et al.*, 1983). The latter observation has been investigated more recently by Tsen and Holtum (2012) and Chomicki and Renner (2016).

In November 1978, Klaus visited Madagascar, sampling Madagascan succulents in the Tsimbazaza Botanic Garden in Tananarive and travelling roughly 750 km to sample in the south-eastern spiny Didiereaceae forests in the vicinity of Fort Dauphin. He noted that the dominance and high biomass of the CAM flora demonstrated that CAM is an ecologically effective photosynthetic process in the semi-arid environments of Madagascar (Winter, 1979). An aspect of the study not mentioned in the succinct paper in *Oecologia* was the difficulty he faced in exporting his plant samples. Madagascar had been recently liberated from colonialism but was virtually bankrupt. Klaus had to convince sceptical customs officials that he was not a smuggler, that the dozens of small dried plant samples were not of medical value and did not represent an attempt to illegally export Madagascar’s sovereign wealth.

## OPPORTUNITY IN NORTH AMERICA

In 1980 Klaus moved to a postdoctoral research position in Gerry Edwards’ laboratory in the Horticulture Department at the University of Wisconsin, Madison. Gerry’s laboratory and Hal Hatch’s laboratory in Canberra were the pre-eminent laboratories investigating C_4_ photosynthesis but Gerry’s laboratory also had interests in the mechanisms of CAM photosynthesis (e.g. Spalding *et al.*, 1979). By coincidence, Klaus was able to continue collaborating with Joe Holtum, who also took up a postdoctoral position at Wisconsin but with Marion O’Leary in the Department of Chemistry.

**Fig. 4.**
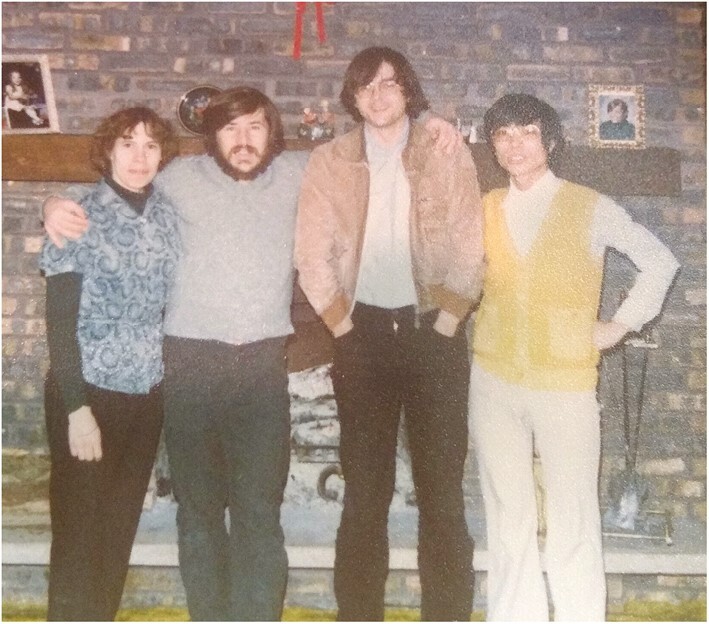
The Madison years, at the home of Gerry and Sandy Edwards, 1980. From left to right: Joyce Foster, Joe Holtum, Klaus Winter and Hitoshi Nakamoto. Photo: G. E. Edwards.

In Madison, Klaus continued his pursuit of the physiology and biochemistry of CAM, producing 12 publications, mostly on *M. crystallinum*, which, by now, was a model species for studies on CAM (Bohnert *et al.*, 1988). Collaborating with Joyce Foster (Fig. 4), Gerry Edwards, Mark Schmitt, Geoff Arron, Tsuzuki Mikio, Hideaki Usuda and Joe Holtum, Klaus published studies that included changes in the level of PEPC during induction of CAM in *M. crystallinum* (Foster *et al.*, 1982); activity and quantity of PEPC and Rubisco in relation to leaf age and nitrogen nutrition during a day–night cycle in *M. crystallinum* and *Kalanchoë pinnata* (Winter *et al.*, 1982*b*); evidence against a previous hypothesis that in *M. crystallinum* nocturnal accumulation of malic acid occurs in mesophyll tissue with proton transport to epidermal tissue (Winter *et al.*, 1981*a*); malate decarboxylation by mitochondrial NAD-malic enzyme in *M. crystallinum* in the CAM mode (Winter *et al.*, 1986*a*); and an investigation of malate metabolism in a non-CAM plant that had been incorrectly identified as CAM (Winter, 1981*b*). It was whilst in Madison that Klaus began publishing non-CAM papers (e.g. Winter *et al.*, 1982*c*, *d*, *e*).

Significantly, it was primarily during his Madison postdoc that Klaus and colleagues established the intracellular localization and activities of CAM enzymes in CAM and C_3_*Mesembryanthemum* (Holtum and Winter, 1982; Tsuzuki *et al.*, 1982; Winter *et al.*, 1982*a*, *d*). This information was used to infer the intracellular movement of carbon during CAM, the enzymes required, their compartmentation, the requirements for exchanges of metabolites via membrane transport, and how the system may be regulated (Edwards *et al.*, 1982; Foster *et al.*, 1983). This biochemical model of CAM was studied, not by surveying gene expression, but by enzymatically digesting leaves to obtain mesophyll protoplasts from C_3_ and CAM tissues. Intracellular compartmentation and the extent of enzyme activities was established by assaying chloroplast, mitochondrial and cytosolic fractions separated following differential and density gradient centrifugation. The product of this research still forms the basis of our understanding of CAM biochemistry (e.g. Winter and Smith, 1996*a*, *b*; Holtum *et al.*, 2005), although we now know that CAM has evolved independently at least 67 times (Gilman *et al.*, 2023) and significant variations in CAM biochemistry occur (e.g. Holtum *et al.*, 2005; Moreno-Villena *et al.*, 2022).

## A RETURN TO GERMANY

In 1981 Klaus returned to Germany under the auspices of a DFG Habilitation Fellowship at the University of Würzburg, where the Botany Department, under the astute leadership of Otto Lange, was an international hub of plant ecophysiological research. The same year he was awarded the prestigious Heinz Maier-Leibnitz Prize, a premier DFG award that recognizes outstanding achievements of early-career researchers and provided a DM 10 000 incentive for support.

Klaus attended the 1982 CAM conference at University of California, Riverside, effectively the first international CAM congress (Ting and Gibbs, 1982), where many prominent CAM researchers from around the world met for the first time and established career-long friendships. For example, at the meeting Klaus first met Clanton Black, Park Nobel and Irwin Ting and re-met Andrew Smith, with whom he has frequently published and from whom he has received much sound advice on the vagaries of written English. Among my many memories of the congress is one of a middle-aged Professor Manfred Kluge, dressed in a suit, most perplexed, attempting to convince a bouncer at the local bar that he was indeed over 21 and trying to explain why his German passport, which was all he had to proffer as ID, did not include a local Californian address!

On completion of his habilitation (Dr. rer. nat. habil.) in 1983, Klaus won a 5-year Heisenberg Fellowship, a DFG award for up-and-coming researchers who had published research of high academic quality and originality and who were promising university teachers.

**Fig. 5.**
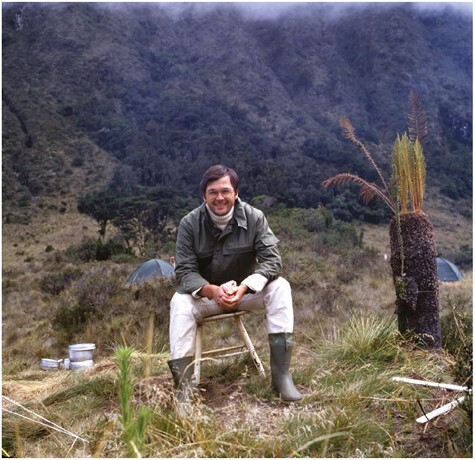
Klaus Winter in the Papua New Guinea Highlands in the vicinity of Mt Wilhelm during the 1984 Oakham School Expedition to Papua New Guinea. Photo: K. Winter.

Whilst at Würzburg, Klaus maintained his interest in the tropical diversity of CAM by participating in the 1984 Oakham School Expedition to Papua New Guinea (Fig. 5). The expedition enabled Klaus to survey succulent epiphytes along an altitudinal gradient from Lae on the eastern coast through the Chimbu Valley, Kegslug and Kundiawa, to the timberline of Mt Wilhelm (4509 m), the highest mountain in PNG. Thirty-five years later, the resulting manuscript (Earnshaw *et al.*, 1987) remains the only CAM study of the flora of New Guinea, the island with the highest plant diversity of any island on Earth (Camara-Leret *et al.*, 2020). In terms of CAM plants, it is estimated that the island supports in excess of 3000 species of orchids, 100 species of *Hoya* and *Dischidia*, and an essentially unassessed diversity of ant-plants (Simonsson Juhonewe and Rodda, 2017; de Vogel *et al.*, 2022).

In Würzburg, Klaus began a fruitful collaboration with Barbara Demmig, a PhD student in Wurzburg who, in 1984, undertook a postdoc with Olle Björkman at the Carnegie Institute at Stanford before returning to Wurzburg in 1986 to undertake her habilitation. Their initial publications were on the ionic, osmotic and photoprotective nature of chloroplasts of CAM plants, particularly *M. crystallinum* in both C_3_ and CAM modes (Demmig and Winter, 1983*a*, *b*, 1986; Winter and Demmig, 1987).

1984 was the only year during his career in which Klaus did not publish. The reason was that he spent much of 1984 researching and writing a detailed synthesis of CAM plant ecophysiology, which was published the following year (Winter, 1985), the year he was appointed Professor of Botany (C3) at Würzburg. Between 1985 and 1987, in a series of mainly collaborative ecophysiological and physiological papers, topics from within his review were explored: CAM in the roots of leafless orchids was demonstrated, in effect following up on Klaus’ isotopic observations of Australian leafless orchids (Winter *et al.*, 1983, 1985); and stomatal patterns and nocturnal acidification in *Welwitschia mirabilis* (a gnetophyte) were addressed (Winter and Schramm, 1986; von Willert *et al.*, 2005). Also explored were the responses of CAM plants that grew under low light conditions (Winter *et al.*, 1986*b*), gradients of CAM within leaves of *Kalanchoë daigremontiana* (Winter, 1987), the contribution of respiratory CO_2_ as carbon source for nocturnal acid synthesis at high temperatures (Winter *et al.*, 1986*c*), and the relationship between turgor pressure and tissue acidity (Rygol *et al.*, 1987).

Twenty-five publications between 1988 and 1991 were primarily non-CAM collaborations with Demmig and colleagues on chlorophyll fluorescence, the xanthophyll cycle, photoinhibition and photoprotection. Two CAM-related studies of South and Central American plants, a study of photoprotection in Venezuelan cacti (Adams *et al.*, 1989) and an investigation of the xanthophyll cycle in *Clusia rosea* (Winter *et al.*, 1990; his first *Clusia* publication), were perhaps a harbinger of a major change in his career that was about to occur, a move from Würzburg to Panama.

## THE PANAMA YEARS

In 1991, Klaus took up a staff scientist position at the Smithsonian Tropical Research Institute (STRI) in the Republic of Panama. In addition to being well-funded, STRI offers strong technical support and provides multiple locations for field research, to include a lowland rainforest on Barro Colorado Island, a montane rainforest at Fortuna, Panama, and two sites with canopy cranes, one in an Atlantic coast forest and the other in a coastal Pacific forest. The move from a research and teaching position in Würzburg to an predominantly research position at STRI surprised some European colleagues but was certainly not out of character. Effectively his new job was to study tropical plant ecophysiology and to communicate. Of course, although not specifically employed to investigate CAM plants, many CAM plants are plants of the tropics!

**Fig. 6.**
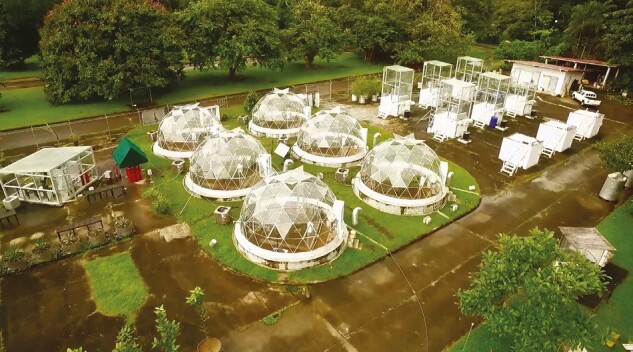
Winter’s open-air laboratory, ‘Winterland’, the STRI Gamboa Research Station with climate-controlled domes, CO_2_-controlled enclosed chambers, open-top chambers, tree-sized pots and the *Clusia* collection. Photo: M. Garcia.

After three decades, Klaus remains at STRI, having been promoted to senior staff scientist in 1998. His outstanding performance has been supported by collaboration with a series of talented graduate students, postdocs and visitors, and by the availability of research infrastructure at STRI, particularly the acquisition in 1993 of land adjacent to the Panama Canal at Gamboa, of which 1 ha was developed into a plant ecophysiology research site. The space, affectionately known as ‘Winterland’ to the international CAM community, is home to the Tropical Domes project (Fig. 6). The domes provide replicate controlled experimental spaces within which plants can be grown in soil under altered concentrations of CO_2_ (or other gases), temperatures and water supplies.

**Fig. 7.**
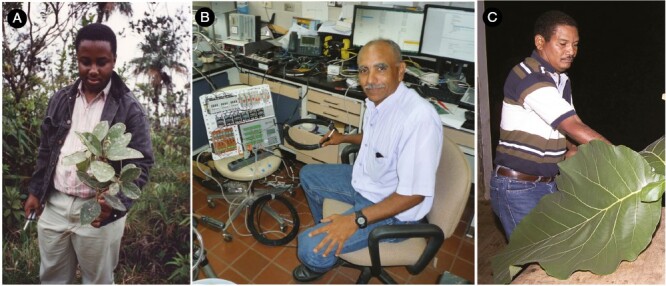
The engine-room of the STRI Winter laboratory: Dr Aurelio Virgo in the cloud forest at Cerro Jefa, Panama (A), Milton Garcia the technical whiz (B) and Jorge Aranda processing a leaf of *Tectona grandis* (teak) (C). Photos: K. Winter (A and B) and M. Guerra (C).

**Fig. 8.**
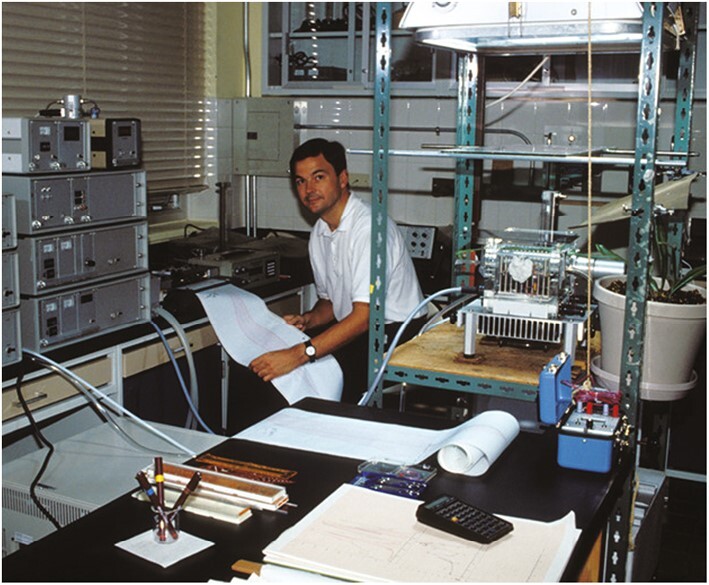
For his first 20 years at STRI, Klaus produced hundreds of metres of chart paper to record plant gas-exchange results, all of which was patiently transcribed to digital values by hand. Photo: K. Winter.

The high productivity of the Winter laboratory has been significantly accelerated by the expertise of the long-serving ‘engine room’ of Dr Aurelio Virgo, Milton Garcia and Jorge Aranda (Fig. 7a–c), who have cultured plants, constructed and maintained and updated physical and physiological infrastructure (such as enabling the transfer from paper to digital recording of gas-exchange output; Fig. 8) and undertaken laboratory analyses (including thousands of acid titrations). Together, they have been co-authors on 44 articles with Klaus.

**Fig. 9.**
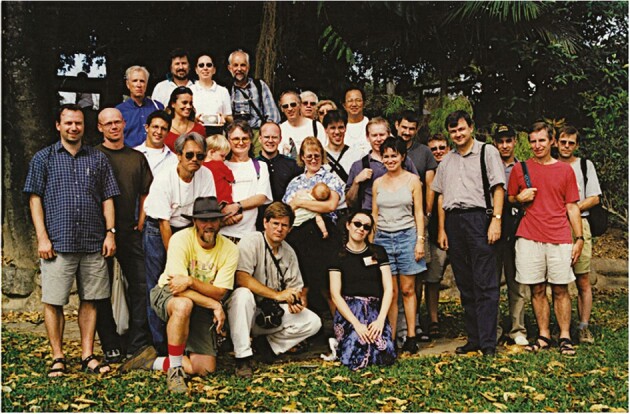
Attendees of CAM: 2001, held amongst the rainforest of Cape Tribulation in northern Queensland, Australia. From left to right: Front row: Lonnie Guralnik, Rowan Sage and Jennifer Henry. Second tier: Stefan Arndt, Uwe Rascher, Maik Zabrowski, Hideaki Usuda, Jack and Nicholas Christopher, Mark Schöttler, Mandy and Ruby Christopher, James Hartwell, Andrew Smith, Anne Borland, Wolfgang Wanek, Jeremy Barnes, Klaus Winter, Tahar Taybi, Howard Griffiths and Jiri Santrucek. Back tier: Dieter von Willert, Fernanda Reinert, Joe Holtum, Kate Maxwell, Ulrich Lüttge, Hugh Nimmo, Bill Cockburn, Olga Cockburn and Akihira Nose. Photo: J. Henry.

**Fig. 10.**
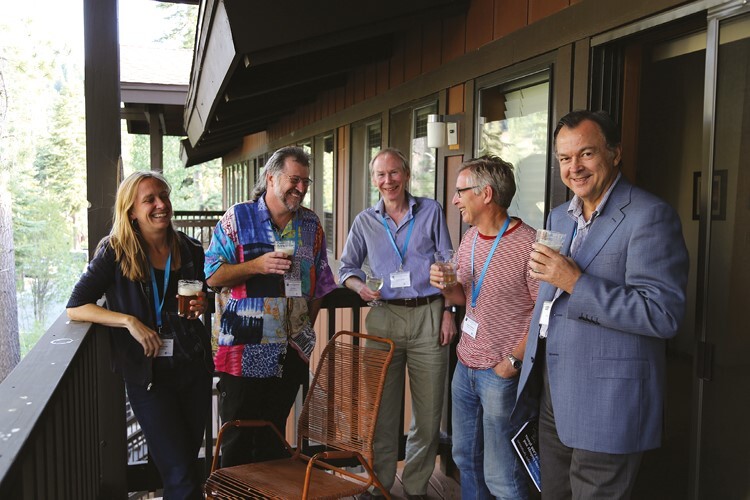
Rehydrating during the 34th New Phytologist Symposium on Systems Biology and Ecology of CAM Plants, Lake Tahoe, 2014. From left to right: Erika Edwards, Joe Holtum, Andrew Smith, Howard Griffiths and Klaus Winter. Photo: R. F. Sage.

The infrastructure and human resource support at STRI has enabled Klaus to investigate not just CAM but to expand his interests in the more general ecophysiology of climate change, CO_2_ effects, water relations, light, temperature and nutrients, and functional traits (for example, Winter, 2023). Of ~209 articles Klaus authored or co-authored at STRI, about 92 (44 %) have been on CAM. STRI support also enabled Klaus to promote CAM networking, graduate training and information exchange by hosting international CAM congresses in 1993, 2010 and March 2023. Support from STRI also fostered scientific productivity by allowing students and colleagues to spend appreciable time in Panama. Klaus’ support was also provided through active participation in international CAM meetings at Cape Tribulation (Fig. 9), Cambridge (2007), Merida (2012), Illinois (2013), Lake Tahoe (2004, 2014) and Phoenix (2018) (Figs 9 and 10).

**Fig. 11.**
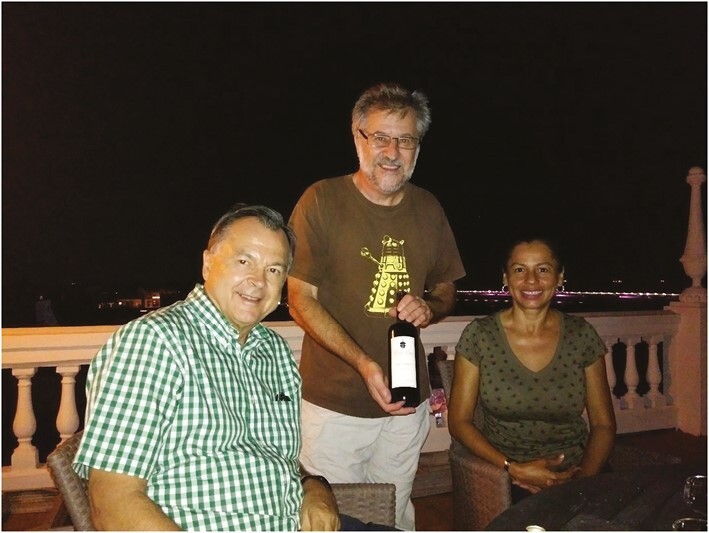
Klaus Winter, Joe Holtum and Noris Arrocha enjoying the fruits of photosynthesis and contemplating the next 50 years on a rooftop balcony during a balmy tropical evening at San Felipe, Panama City, in 2018. Photo: J. A. M. Holtum.

At STRI, Klaus continues to publish with long-term CAM colleagues (Table 1), particularly with Joe Holtum and Andrew Smith (Fig. 10). Since their first publication (Winter *et al.*, 1981*a*), Winter and Holtum have co-authored 39 papers, almost all on CAM, of which 35 have been published since Klaus arrived at STRI (see https://stri-sites.si.edu/docs/cvs/CV_Klaus_Winter_Oct_2022.pdf).

**Table 1.** Authors with whom, whilst at STRI, Klaus Winter has collaborated most frequently on CAM topics. Exemplar references are provided. Information summarized from https://stri-sites.si.edu/docs/cvs/CV_Klaus_Winter_Oct_2022.pdf

Collaborator	Institution	Topics and number of joint publications		Example publication
Joseph Holtum	James Cook University, Australia	CAM discovery, facultative CAM	35	Winter *et al.*, 1981*a*
Andrew Smith	University of Oxford, UK	CAM cell physiology and evolution	19	Winter and Smith, 2022
John Cushman	University of Nevada-Reno, USA	Evolutionary biology of CAM	9	Yang *et al.*, 2017
Erika Edwards	Yale University, USA	Evolution of CAM, CAM in Caryophyllales	8	Winter *et al.*, 2019
Katja Silvera	STRI/University of California, USA	Evolution of CAM in orchids	8	Silvera *et al.*, 2014
Darren Crane	STRI/James Cook University, Australia	CAM within Bromeliaceae	7	Crayn *et al.*, 2015
Anne Borland	Newcastle University, UK	CAM evolution and water relations	6	Leverett *et al.*, 2023
Howard Griffiths	University of Cambridge, UK	CAM water relations	6	Mejia-Chang *et al.*, 2021
Hans Gehrig	STRI, Panama	CAM PEPC and evolution of *Clusia*	4	Gehrig *et al.*, 2005
John Skillman	California State University, USA	CAM in the understorey	4	Skillman *et al.*, 1999
Simon Pierce	STRI/University of Milan, Italy	Ecophysiology of bromeliads	3	Pierce *et al.*, 2002

An important event in CAM science was the publication in 1996 of a monograph on CAM in the Springer Ecological Studies series (Winter and Smith, 1996*a*). The monograph, which had its genesis during the CAM conference held at STRI in Panama in 1993, documented the 18 years of CAM research since the previous CAM monograph (Kluge and Ting, 1978). Notably, the 1996 book included the first list of known CAM plants (Smith and Winter, 1996), 328 genera in 33 families at the time, and a major summative chapter that reconciled contrasting energetic costings for CAM (Winter and Smith, 1996*b*). The updated list of plants with CAM published in this volume (Gilman *et al.*, 2023), which includes many name changes and newly recognized C_3_-CAM and facultative species, conservatively indicates at least 67 independent origins of CAM in 320+ genera in 38 families.

An important facet of Klaus’s tenure at STRI has been an expansion of his scientific footprint from mainly CAM research to examining interactions between tropical plant ecophysiology and climate change. With STRI postdocs, staff and visitors, he has explored in the field and/or under controlled conditions the responses of tropical angiosperms, conifers, pioneer and late-successional species, epiphytes, mangroves and mycorrhiza to soil nutrients, low and high [CO_2_], high irradiation, high temperature, water availability, photoinhibition and ozone levels (Table 2).

**Table 2.** Authors with whom, whilst at STRI, Klaus Winter has collaborated on non-CAM topics of tropical plant ecophysiology. Exemplar publications are provided. Information summarized from https://stri-sites.si.edu/docs/cvs/CV_Klaus_Winter_Oct_2022.pdf

Collaborator	Institution	Topics and number of joint publications		Example publication
Ben Turner	STRI, Panama	Effects of nutrition/climate change on trees	24	Nasto *et al.*, 2019
Heinrich Krause	University of Düsseldorf, FRG	Tropical photosynthesis under high irradiance and temperature stress	23	Krause and Winter, 2021
Martijn Slot	STRI, Panama	Tropical tree functional responses to climate change	19	Slot *et al.*, 2021*a*, *b*, *c*
Lucas Cernusak	STRI/James Cook University, Australia	Effects of climate change, nutrients, high [CO_2_] on tropical angiosperms and conifers	15	Cernusak *et al.*, 2008, 2009, 2011, 2013
Gerhard Zotz	STRI/University of Oldenberg, FRG	Tropical tree epiphytes and hemiepiphytes	12	Zotz and Winter, 1996
Cathy Lovelock	STRI/University of Queensland, Australia	Effect of elevated CO_2_ on mangroves and mycorrhiza	11	Lovelock *et al.*, 1997
Jim Dalling	STRI/University of Illinois, USA	Tropical angiosperms and gymnosperm responses to [CO_2_], nutrient levels and water availability	7	Dalling *et al.*, 2004
Marianne Popp	University of Vienna, Austria	Responses on tropical vegetation to elevated CO_2_	6	Lovelock *et al.*, 1998
Alex Cheesman	STRI/James Cook University, Australia	Responses to ozone, nutrients and temperature	6	Schneider *et al.*, 2017
Ruth Reef	STRI/Monash University, Australia	Effects of CO_2_ and environment on mangroves	2	Reef *et al.*, 2016

For 50 years Klaus has been an indefatigable experimentalist, providing plant environmental and photosynthetic ecophysiology data for more than 300 publications. The driver of this productivity is Klaus’ natural and perhaps somewhat obsessive curiosity to understand how plants function in the real world. For example, although Klaus reported inducible CAM in *M. crystallinum* in his first publication (Winter and von Willert, 1972) it was not until 2007 that he could demonstrate experimentally that the expression of CAM in *M. crystallinum* was truly under environmental control (Winter and Holtum, 2007). To establish the latter he had to use a career’s experience of performing gas exchange to grow *M. crystallinum* from seed to death without the plants ever performing the net nocturnal CO_2_ uptake typical of CAM. Experimental skills initially learnt and honed investigating CAM ecophysiology as a student and in his early postdoc years were adapted at STRI to examine the wider world of plant responses to climate change, particularly the readjustments of tropical vegetation. Although trained in a pre-molecular era, he has embraced the molecular revolution, particularly in systematics and phylogenetics (e.g. Brilhaus *et al.*, 2016; Ávila-Lovera *et al.*, 2022; Lujan *et al.*, 2022).

The large number of scientists who have collaborated with him, and continue to do so, attests to the facilities he has constructed, his experimental skills, his potent intellect, his creativity in designing experiments to test questions, his amenable nature and his positive attitude to collaborative research and sharing knowledge. For a more personal insight into Klaus Winter’s career the reader should visit ‘Brief reflections on 50 years as a plant ecophysiologist’ (Winter, 2023).

## References

[CIT0001] Adams WW , Diaz M, Winter K. 1989. Diurnal changes in photochemical efficiency, the reduction state of Q, radiationless energy dissipation, and non-photochemical fluorescence quenching in cacti exposed to natural sunlight in northern Venezuela. Oecologia 80: 553–561. doi:10.1007/BF00380081. 28312843

[CIT0002] Atwell B , et al. 2021. Hank Greenway – an inspiring life (1926–2021). *Phytogen*. https://www.asps.org.au/archives/author/drkoerber/page/3. Accessed October 31, 2023.

[CIT0003] Ávila-Lovera E , Winter K, Goldsmith GR. 2022. Evidence for phylogenetic signal and correlated evolution in plant–water relation traits. New Phytologist 237: 392–407. doi:10.1111/nph.18565. 36271615

[CIT0004] Black CC , Osmond CB. 2003. Crassulacean acid metabolism photosynthesis: ‘working the night shift’. Photosynthesis Research 76: 329–341. doi:10.1023/A:1024978220193. 16228591

[CIT0005] Bohnert HJ , Ostrem JA, Cushman JC, et al. 1988. *Mesembryanthemum crystallinum*, a higher plant model for the study of environmentally induced changes in gene expression. Plant Molecular Biology Reporter 6: 10–28. doi:10.1007/bf02675305.

[CIT0006] Brilhaus D , Bräutigam A, Mettler-Altmann T, Winter K, Weber A. 2016. Reversible burst of transcriptional changes during induction of crassulacean acid metabolism (CAM) in *Talinum triangulare*. Plant Physiology 170: 102–122. 26530316 10.1104/pp.15.01076 PMC4704576

[CIT0007] Camara-Leret R , Frodin DG, Adema F, et al. 2020. New Guinea has the world’s richest island flora. Nature 584: 579–583. doi:10.1038/s41586-020-2549-5. 32760001

[CIT0008] Cernusak LA , Winter K, Aranda J, Turner BL. 2008. Conifers, angiosperm trees, and lianas: growth, whole-plant water and nitrogen-use efficiency, and stable isotope composition (δ^13^C and δ^18^O) of seedlings grown in a tropical environment. Plant Physiology 148: 642–659. doi:10.1104/pp.108.123521. 18599645 PMC2528101

[CIT0009] Cernusak LA , Winter K, Turner BL. 2009. Plant δ^14^N correlates with transpiration efficiency and nitrogen acquisition in tropical trees. Plant Physiology 151: 1667–1676. doi:10.1104/pp.109.145870. 19726571 PMC2773072

[CIT0010] Cernusak LA , Winter K, Martinez C, et al. 2011. Responses of legume versus nonlegume tropical tree seedlings to elevated CO_2_ concentration. Plant Physiology 157: 372–385. doi:10.1104/pp.111.182436. 21788363 PMC3165885

[CIT0011] Cernusak LA , Ubierna N, Winter K, Holtum JAM, Marshall JD, Farquhar GD. 2013. Environmental and physiological determinants of carbon isotope discrimination in terrestrial plants. New Phytologist 200: 950–965. doi:10.1111/nph.12423. 23902460

[CIT0012] Chomicki G , Renner S. 2016. Evolutionary relationships and biogeography of the ant-epiphytic genus *Squamellaria* (Rubiaceae: Psychotrieae) and their taxonomic implications. PLoS One 11: e0151317. 27028599 10.1371/journal.pone.0151317 PMC4814088

[CIT0013] Coutinho LM. 1963. Algumas informações sôbre a ocorrência do ‘efeito de De Saussure’ em epífitas e erbáceas terrestres da mata pluvial. Boletim da Faculdade de Filosofia, Ciências e Letras, Universidade de São Paulo. Botânica 20: 83–101.

[CIT0014] Crayn DM , Winter K, Schulte K, Smith JAC. 2015. Photosynthetic pathways in Bromeliaceae: phylogenetic and ecological significance of CAM and C_3_ based on carbon isotope ratios for 1893 species. Botanical Journal of the Linnean Society 178: 169–221. doi:10.1111/boj.12275.

[CIT0015] Dalling JW , Winter K, Hubbell SP. 2004. Variation in growth responses of neotropical pioneers to simulated forest gaps. Functional Ecology 18: 725–736. doi:10.1111/j.0269-8463.2004.00868.x.

[CIT0016] de Vogel EF , Vermeulen JJ, Schuiteman A. 2022. *Orchids of New Guinea*. www.orchidsnewguinea.com. Accessed October 21, 2023.

[CIT0017] Demmig B , Winter K. 1983a. Chloroplasts from *Mesembryanthemum crystallinum* L., a halophilic plant capable of crassulacean acid metabolism. Hoppe-Seyler’s Zeitschrift für Physiologische Chemie 364: 1115–1116.

[CIT0018] Demmig B , Winter K. 1983b. Photosynthetic characteristics of chloroplasts from *Mesembryanthemum crystallinum* L., a halophilic plant capable of crassulacean acid metabolism. Planta 159: 66–76. doi:10.1007/BF00998816. 24258088

[CIT0019] Demmig B , Winter K. 1986. Sodium, potassium, chloride and proline concentrations of chloroplasts isolated from a halophyte, *Mesembryanthemum crystallinum* L. Planta 168: 421–426. doi:10.1007/BF00392371. 24232155

[CIT0020] Earnshaw MJ , Winter K, Ziegler H, et al. 1987. Altitudinal changes in the incidence of crassulacean acid metabolism in vascular epiphytes and related life forms in Papua New Guinea. Oecologia 73: 566–572. doi:10.1007/BF00379417. 28311975

[CIT0021] Edwards GE , Foster JG, Winter K. 1982. Activity and intracellular compartmentation of enzymes of carbon metabolism in CAM plants. In: Ting IP, Gibbs M. eds. Crassulacean acid metabolism. Rockville: American Society of Plant Physiologists, 92–111.

[CIT0022] Foster JG , Edwards GE, Winter K. 1982. Changes in levels of phosphoenolpyruvate carboxylase with induction of crassulacean acid metabolism in *Mesembryanthemum crystallinum* L. Plant and Cell Physiology 23: 585–594.

[CIT0023] Foster JG , Edwards GE, Winter K. 1983. Regulation of carbon metabolism in *Mesembryanthemum crystallinum*. In: Marcelle R, Clijsters H, van Poucke M. eds. Effects of stress on photosynthesis. The Hague: Junk, 175–183.

[CIT0024] Gehrig HH , Wood JA, Cushman MA, Virgo A, Cushman JC, Winter K. 2005. Large gene family of phosphoenolpyruvate carboxylase in the crassulacean acid metabolism plant *Kalanchoe pinnata* (Crassulaceae) characterized by partial cDNA sequence analysis. Functional Plant Biology 32: 467–447. doi:10.1071/fp05079. 32689147

[CIT0025] Gilman IS , Smith JAC, Holtum JAM, Sage RF, Winter K, Edwards EJ. 2023. The CAM lineages of planet Earth. Annals of Botany 132: 627–654. 10.1093/aob/mcad135 PMC10799995 37698538

[CIT0026] Greenway H , Winter K, Lüttge U. 1978. Phosphoenolpyruvate carboxylase during development of crassulacean acid metabolism and during a diurnal cycle in *Mesembryanthemum crystallinum*. Journal of Experimental Botany 29: 547–559. doi:10.1093/jxb/29.3.547.

[CIT0027] Haberlandt G. 1884. Physiologische Pflanzenanatomie. Leipzig: Wilhelm Engelmann.

[CIT0028] Hancock LP , Obbens F, Moore AJ, et al. 2018. Phylogeny, evolution, and biogeographic history of *Calandrinia* (Montiaceae). American Journal of Botany 105: 1021–1034. 29995314 10.1002/ajb2.1110

[CIT0029] Hancock LP , Holtum JAM, Edwards EJ. 2019. The evolution of a spectrum of CAM phenotypes in Australian *Calandrinia* (Montiaceae). Integrative and Comparative Biology 59: 517–534. doi:10.1093/icb/icz089. 31161205

[CIT0030] Hattersley PW , Watson L. 1976. C_4_ grasses: an anatomical criterion for distinguishing between NADP-malic enzyme species and PCK or NAD-malic enzyme species. Australian Journal of Botany 24: 297–308. doi:10.1071/bt9760297.

[CIT0031] Holtum JAM , Winter K. 1982. Activity of enzymes of carbon metabolism during the induction of crassulacean acid metabolism in *Mesembryanthemum crystallinum*. Planta 155: 8–16. doi:10.1007/BF00402925. 24271620

[CIT0032] Holtum JA , Smith JAC, Neuhaus HE. 2005. Intracellular transport and pathways of carbon flow in plants with crassulacean acid metabolism. Functional Plant Biology 32: 429–449. 32689145 10.1071/FP04189

[CIT0033] Holtum JAM , Hancock LP, Edwards EJ, et al. 2016. Is the Australian flora depauperate in plants with crassulacean acid metabolism (CAM)? Current Opinion in Plant Science 31: 109–117. 10.1016/j.pbi.2016.03.018 27088716

[CIT0034] Kluge M , Ting IP. 1978. Crassulacean acid metabolism. Analysis of an ecological adaptation. Berlin: Springer.

[CIT0035] Krause GH , Winter K. 2021. The photosynthetic system in tropical plants under high irradiance and temperature stress. Progress in Botany 82: 131–170.

[CIT0036] Leverett A , Ferguson K, Winter K, Borland AM. 2023. Leaf vein density correlates with crassulacean acid metabolism but not hydraulic capacitance, in the genus *Clusia*. Annals of Botany. doi:10.1093/aob/mcad035. PMC10799986 36821473

[CIT0037] Lovelock CE , Kyllo D, Popp M, Isopp H, Virgo A, Winter K. 1997. Symbiotic vesicular-arbuscular mycorrhizae influence maximum rates of photosynthesis in tropical tree seedlings grown under elevated CO_2_. Australian Journal of Plant Physiology 24: 185–194.

[CIT0038] Lovelock CE , Winter K, Mersits R, Popp M. 1998. Responses of communities of tropical tree species to elevated CO_2_. Oecologia 116: 207–218. doi:10.1007/s004420050581. 28308528

[CIT0039] Lujan M , Oleas N, Winter K. 2022. Evolutionary history of CAM photosynthesis in neotropical *Clusia*: insights from genomics, anatomy, physiology and climate. Botanical Journal of the Linnean Society 199: 538–556.

[CIT0040] McWilliams E. 1970. Comparative rates of dark CO_2_ uptake and acidification in the Bromeliaceae, Orchidaceae and Euphorbiaceae. Botanical Gazette 131: 285–290.

[CIT0041] Medina E , Troughton JH. 1974. Dark CO_2_ fixation and the carbon isotope ratio in Bromeliaceae. Plant Science Letters 2: 357–362.

[CIT0042] Mejia-Chang M , Reyes-Garcia C, Seibt U, et al. 2021. Leaf water δ^18^O reflects water vapour exchange and uptake by C_3_ and CAM epiphytic bromeliads in Panama. Functional Plant Biology 48: 732–742. doi:10.1071/fp21087. 34099101

[CIT0043] Moreno-Villena JJ , Zhou H, Gilman IS, Tausta SL, Cheung CM, Edwards EJ. 2022. Spatial resolution of an integrated C_4_+ CAM photosynthetic metabolism. Science Advances 8: eabn2349. 35930634 10.1126/sciadv.abn2349 PMC9355352

[CIT0044] Nasto MK , Winter K, Turner BL, Cleveland CC. 2019. Nutrient acquisition strategies enable high growth rates in tropical nitrogen fixing trees in nutrient poor soil and under elevated carbon dioxide. Ecology 100: e02646. doi:10.1002/ecy.2646. 30714149

[CIT0045] Neales TF , Hew CS. 1975. Two types of carbon fixation in tropical orchids. Planta 123: 303–306. doi:10.1007/BF00390710. 24435130

[CIT0046] Nuernbergk EL. 1961. Endogener Rhythmus und CO_2_-Stoffwechsel bei Pflanzen mit diurnalem Säurerhythmus. Planta 56: 28–70.

[CIT0047] Osmond CB. 1995. To change the way we think about things. In: Shearmur PM, Osmond CB, Pockley P. eds. Nurturing creativity in research: ideas as the foundation of innovation. Canberra: Research School of Biological Sciences, 3–11.

[CIT0048] Osmond CB. 1978. Crassulacean acid metabolism: a curiosity in context. Annual Review of Plant Physiology 9: 379–414.

[CIT0049] Osmond CB. 2014. Our eclectic adventures in the slower eras of photosynthesis: from New England Down Under to Biosphere 2 and beyond. Annual Review of Plant Biology 65: 1–32. 10.1146/annurev-arplant-050213-035739 24779995

[CIT0050] Osmond CB , Allaway WG, Sutton BG, et al. 1973. Carbon isotope discrimination in photosynthesis of CAM plants. Nature 246: 41–42. doi:10.1038/246041a0.

[CIT0051] Pachon P , Winter K, Lasso E. 2022. Updating the occurrence of crassulacean acid metabolism (CAM) in the genus *Clusia* through carbon isotope analysis of species from Colombia. Photosynthetica 60: 304–322. doi:10.32615/ps.2022.018. PMC11558512 39650755

[CIT0052] Pierce S , Winter K, Griffiths H. 2002. The role of CAM in high rainfall cloud forests: an in situ comparison of photosynthetic pathways in Bromeliaceae. Plant Cell Environment 25: 1183–1192.

[CIT0053] Reef R , Slot M, Motro U, et al. 2016. The effects of CO_2_ and nutrient fertilisation on the growth and temperature response of the mangrove *Avicennia germinans*. Photosynthesis Research 129: 159–170. doi:10.1007/s11120-016-0278-2. 27259536

[CIT0054] Rygol J , Winter K, Zimmermann U. 1987. Relationship between turgor pressure and tissue acidity in mesophyll cells of intact leaves of a crassulacean acid metabolism plant, *Kalanchoe daigremontiana* Hamet et Perr. Planta 142: 487–493. 10.1007/BF00393864 24226067

[CIT0055] Schneider GF , Cheesman AW, Winter K, Turner BL, Sitch S, Kursar TA. 2017. Current ambient concentrations of ozone in Panama modulate the leaf chemistry of the tropical tree *Ficus insipida*. Chemosphere 172: 363–372. doi:10.1016/j.chemosphere.2016.12.109. 28088026

[CIT0056] Silvera K , Winter K, Rodriguez BL, Albion RL, Cushman JC. 2014. Multiple isoforms of phosphoenolpyruvate carboxylase in the Orchidaceae (subtribe Oncidiinae): implications for the evolution of crassulacean acid metabolism. Journal of Experimental Botany 65: 3623–3636. doi:10.1093/jxb/eru234. 24913627 PMC4085970

[CIT0057] Simonsson Juhonewe N , Rodda M. 2017. Contribution to a revision of *Hoya* (Apocynaceae: Asclepiadoideae) of Papuasia. Part I: ten new species, one new subspecies and one new combination. Gardens’ Bulletin Singapore 69: 97–147.

[CIT0058] Skillman JB , Garcia M, Winter K. 1999. Whole-plant consequences of crassulacean acid metabolism for an understory bromeliad in a tropical moist forest; a comparative study. Ecology 80: 1584–1593. doi:10.1890/0012-9658(1999)080[1584:wpcoca]2.0.co;2.

[CIT0059] Slot M , Cala D, Aranda J, Virgo A, Michaletz ST, Winter K. 2021a. Leaf heat tolerance of 147 tropical forest species varies with elevation and leaf functional traits, but not with phylogeny. Plant Cell and Environment 44: 2414–2427. doi:10.1111/pce.14060. 33817813

[CIT0060] Slot M , Nardwattanawong T, Hernández GG, Bueno A, Riederer M, Winter K. 2021b. Large differences in leaf cuticle conductance and its temperature response among 24 tropical tree species from across a rainfall gradient. New Phytologist 232: 1618–1631. doi:10.1111/nph.17626. 34270792 PMC9290923

[CIT0061] Slot M , Rifai S, Winter K. 2021c. Photosynthetic plasticity of a tropical tree species, *Tabebuia rosea*, in response to elevated temperature and [CO_2_]. Plant Cell and Environment 44: 2347–2364. 10.1111/pce.14049 33759203

[CIT0062] Smith JAC , Winter K. 1996. Taxonomic distribution of crassulacean acid metabolism. In: Winter K, Smith JAC. eds. Crassulacean acid metabolism. Berlin: Springer, 427–436.

[CIT0063] Spalding MH , Schmitt MR, Ku SB, Edwards GE. 1979. Intracellular localization of some key enzymes of crassulacean acid metabolism in *Sedum praealtum*. Plant Physiology 63: 738–743. doi:10.1104/pp.63.4.738. 16660803 PMC542908

[CIT0064] Sutton BG. 1975. The path of carbon in CAM plants at night. Functional Plant Biology 2: 377–387. doi:10.1071/pp9750377.

[CIT0065] Ting IP , Gibbs M. 1982. Crassulacean acid metabolism: proceedings of the fifth annual symposium in botany. Riverside, California, January 14-15, 1982. Rockville: American Society of Plant Physiologists.

[CIT0066] Tsen EWJ , Holtum JAM. 2012. Crassulacean acid metabolism (CAM) in an epiphytic ant-plant, *Myrmecodia beccarii* Hook.f. (Rubiaceae). Photosynthesis Research 113: 311–320. doi:10.1007/s11120-012-9732-y. 22442054

[CIT0067] Tsuzuki M , Miyachi S, Winter K, Edwards GE. 1982. Localization of carbonic anhydrase in crassulacean acid metabolism plants. Plant Science Letters 24: 211–218.

[CIT0068] Volkens G. 1887. Die Flora der aegyptisch-arabischen Wüste auf Grundlage anatomisch-physiologischer Forschungen. Berlin: Gerbrüder Borntraeger.

[CIT0069] von Willert DJ , Armbrüster N, Drees T, Zaborowski M. 2005. *Welwitschia mirabilis*: CAM or not CAM – what is the answer? Functional Plant Biology 32: 389–395. doi:10.1071/fp01241. 32689141

[CIT0070] Winter K. 1972. Untersuchungen zum NaCl-induzierten Crassulaceensäurestoffwechsel bei Mesembryanthemum crystallinum. Staatsexamensarbeit: Technische Hochschule Darmstadt.

[CIT0071] Winter K. 1973a. Zum Problem der Ausbildung des Crassulaceen-Säurestoffwechsels bei *Mesembryanthemum crystallinum* unter NaCl-Einfluß. Planta 109: 135–145. doi:10.1007/BF00386121. 24474059

[CIT0072] Winter K. 1973b. CO_2_-Fixierungsreaktionen bei der Salzpflanze *Mesembryanthemum crystallinum* unter variierten Außenbedingungen. Planta 114: 75–85. doi:10.1007/BF00390286. 24458666

[CIT0073] Winter K. 1973c. NaCl-induzierter Crassulaceen-Säurestoffwechsel bei einer weiteren Aizoacee: *Carpobrotus edulis*. Planta 115: 187–188. doi:10.1007/BF00387783. 24458867

[CIT0074] Winter K. 1973d. CO_2_-Gaswechsel von an hohe Salinität adaptiertem *Mesembryanthemum crystallinum* bei Rückführung in glykisches Anzuchtmedium. Berichte der Deutschen Botanischen Gesellschaft 86: 467–476.

[CIT0075] Winter K. 1974a. NaCl-induzierter Crassulaceen-Säurestoffwechsel bei der Salzpflanze *Mesembryanthemum crystallinum*. Abhängigkeit des CO_2_-Gaswechsels von der Tag/Nachttemperatur und von der Wasserversorgung der Pflanzen. Oecologia 15: 383–392. doi:10.1007/BF00345435. 28308633

[CIT0076] Winter K. 1974b. Evidence for the significance of crassulacean acid metabolism as an adaptive mechanism to water stress. Plant Science Letters 3: 279–281.

[CIT0077] Winter K. 1974c. Einfluß von Wasserstreß auf die Aktivität der Phosphoenolpyruvat Carboxylase bei *Mesembryanthemum crystallinum* L. Planta 121: 147–153. doi:10.1007/BF00388753. 24442778

[CIT0078] Winter K. 1974d. Wachstum und Photosyntheseleistung der Halophyten *Mesembryanthemum nodiflorum* L. and *Suaeda maritima* (L.) Dum. bei variierter NaCl- Salinität des Anzuchtmediums. Oecologia 17: 317–324. doi:10.1007/BF00345749. 28308945

[CIT0079] Winter K. 1975. Die Rolle des Crassulaceen-Säurestoffwechsels als biochemische Grundlage zur Anpassung von Halophyten an Standorte hoher Salinität.Dr. rer. nat. Dissertation, Technische Hochschule Darmstadt, Germany.

[CIT0080] Winter K. 1979. δ^13^C Values of succulent plants from Madagascar. Oecologia 40: 103–112. doi:10.1007/BF00388814. 28309607

[CIT0081] Winter K. 1980. Day/night changes in the sensitivity of phosphoenolpyruvate carboxylase to malate during crassulacean acid metabolism. Plant Physiology 65: 792–796. doi:10.1104/pp.65.5.792. 16661284 PMC440426

[CIT0082] Winter K. 1981a. Change in properties of phosphoenolpyruvate carboxylase from the crassulacean acid metabolism plant *Mesembryanthemum crystallinum* after isolation. Functional Plant Biology 8: 115–119. doi:10.1071/pp9810115.

[CIT0083] Winter K. 1981b. CO_2_ and water vapour exchange, malate content and δ^13^C value in *Cicer arietinum*: grown under two water regimes. Zeitschrift für Pflanzenphysiologie 101: 421–430.

[CIT0084] Winter K. 1982. Properties of phosphoenolpyruvate carboxylase in rapidly prepared, desalted leaf extracts of the crassulacean acid metabolism plant *Mesembryanthemum crystallinum* L. Planta 154: 298–308. doi:10.1007/BF00393907. 24276156

[CIT0085] Winter K. 1985. Crassulacean acid metabolism. In: Barber J, Baker NR. eds. Photosynthetic mechanisms and the environment. Amsterdam: Elsevier, 329–387.

[CIT0086] Winter K. 1987. Gradient in the degree of crassulacean acid metabolism within leaves of *Kalanchoe daigremontiana*. Planta 172: 88–90. doi:10.1007/BF00403032. 24225791

[CIT0087] Winter K. 2023. Brief reflections on 50 years as a plant ecophysiologist. Annals of Botany 132: 577–582. 10.1093/aob/mcad020 PMC10799979 36751882

[CIT0088] Winter K , Demmig B. 1987. Reduction state of Q and nonradiative energy dissipation during photosynthesis in leaves of a Crassulacean acid metabolism plant, *Kalanchoë daigremontiana* Hamet et Perr. Plant Physiology 85: 1000–1007. doi:10.1104/pp.85.4.1000. 16665793 PMC1054383

[CIT0089] Winter K , Greenway H. 1978. Phosphoenolpyruvate carboxylase from *Mesembryanthemum crystallinum*: its isolation and inactivation in vitro. Journal of Experimental Botany 29: 539–546. doi:10.1093/jxb/29.3.539.

[CIT0090] Winter K , Holtum JAM. 2007. Environment or development? Lifetime net CO_2_ exchange and control of the expression of crassulacean acid metabolism in *Mesembryanthemum crystallinum*. Plant Physiology 143: 98–107. doi:10.1104/pp.106.088922. 17056756 PMC1761986

[CIT0091] Winter K , Holtum JAM. 2011. Induction and reversal of crassulacean acid metabolism in *Calandrinia polyandra*: effects of soil moisture and nutrients. Functional Plant Biology 38: 576–582. doi:10.1071/fp11028. 32480910

[CIT0092] Winter K , Schramm MJ. 1986. Analysis of stomatal and nonstomatal components in the environmental control of CO_2_ exchange in leaves of *Welwitschia mirabilis*. Plant Physiology 82: 173–178. doi:10.1104/pp.82.1.173. 16664987 PMC1056085

[CIT0093] Winter K , Smith JAC. 1996a. Crassulacean acid metabolism. Biochemistry, ecophysiology and evolution. Berlin: Springer.

[CIT0094] Winter K , Smith JAC. 1996b. Crassulacean acid metabolism: current status and perspectives. In: Winter K, Smith JAC. eds. Crassulacean acid metabolism. Biochemistry, ecophysiology and evolution. Berlin: Springer, 389–426.

[CIT0095] Winter K , Smith JAC. 2022. CAM photosynthesis: the acid test. New Phytologist 233: 599–609. 34637529 10.1111/nph.17790 PMC9298356

[CIT0096] Winter K , Troughton JH. 1978a. Photosynthetic pathways in plants of coastal and inland habitats of Israel and the Sinai. Flora 167: 1–34. doi:10.1016/s0367-2530(17)31087-3.

[CIT0097] Winter K , Troughton JH. 1978b. Carbon assimilation pathways in *Mesembryanthemum nodiflorum* L. under natural conditions. Zeitschrift für Pflanzenphysiologie 88: 153–162.

[CIT0098] Winter K , von Willert DJ. 1972. NaCl-induzierter Crassulaceensäurestoffwechsel bei *Mesembryanthemum crystallinum*. Zeitschrift für Pflanzenphysiologie 67: 166–170.

[CIT0099] Winter K , Lüttge U, Ball E. 1974. ^14^CO_2_ dark fixation in the halophytic species *Mesembryanthemum crystallinum*. Biochimica et Biophysica Acta 343: 465–468. doi:10.1016/0304-4165(74)90263-3. 4407030

[CIT0100] Winter K , Troughton JH, Card KA. 1976a. δ^13^C values of grass species collected in the northern Sahara Desert. Oecologia 25: 115–123. doi:10.1007/BF00368848. 28308994

[CIT0101] Winter K , Troughton JH, Evenari M, Läuchli A, Lüttge U. 1976b. Mineral ion composition and occurrence of CAM like diurnal malate fluctuations in plants of coastal and desert habitats of Israel and the Sinai. Oecologia 25: 125–143. doi:10.1007/BF00368849. 28308995

[CIT0102] Winter K , Kramer D, Troughton JH, Card KA, Fischer K. 1977. C_4_ pathway of photosynthesis in a member of the Polygonaceae: *Calligonum persicum* (Boiss. & Buhse) Boiss. Zeitschrift für Pflanzenphysiologie 81: 341–346.

[CIT0103] Winter K , Lüttge U, Winter E, Troughton JH. 1978. Seasonal shift from C_3_ photosynthesis to crassulacean acid metabolism in *Mesembryanthemum crystallinum* growing in its natural environment. Oecologia 34: 225–237. doi:10.1007/BF00345168. 28309551

[CIT0104] Winter K , Edwards GE, Holtum JAM. 1981a. Nocturnal accumulation of malic acid occurs in mesophyll tissue without proton transport to epidermal tissue in the inducible crassulacean acid metabolism plant *Mesembryanthemum crystallinum*. Plant Physiology 68: 355–357. doi:10.1104/pp.68.2.355. 16661916 PMC427490

[CIT0105] Winter K , Osmond CB, Pate JS. 1981b. Coping with salinity. In: Pate JS, McComb AJ. eds. The biology of Australian plants. Nedlands: University of Western Australia Press, 88–113.

[CIT0106] Winter K , Foster JG, Edwards GE, Holtum JAM. 1982a. Intracellular localization of enzymes of carbon metabolism in *Mesembryanthemum crystallinum* exhibiting C_3_ photosynthetic characteristics or performing crassulacean acid metabolism. Plant Physiology 69: 300–307. doi:10.1104/pp.69.2.300. 16662197 PMC426198

[CIT0107] Winter K , Foster JG, Schmitt MS, Edwards GE. 1982b. Activity and quantity of ribulose bisphosphate carboxylase- and phosphoenolpyruvate carboxylase-protein in two crassulacean acid metabolism plants in relation to leaf age, nitrogen nutrition and point in time during a day/night cycle. Planta 154: 309–317. 24276157 10.1007/BF00393908

[CIT0108] Winter K , Holtum JAM, Edwards GE, O’Leary MH. 1982c. Effect of low relative humidity on δ^13^C value in two C_3_ grasses and in *Panicum milioides*, a C_3_-C_4_ intermediate species. Journal of Experimental Botany 33: 88–91. doi:10.1093/jxb/33.1.88.

[CIT0109] Winter K , Usuda H, Tsuzuki M, et al. 1982d. Influence of nitrate and ammonia on photosynthetic characteristics and leaf anatomy of *Moricandia arvensis*. Plant Physiology 70: 616–625. doi:10.1104/pp.70.2.616. 16662544 PMC1067198

[CIT0110] Winter K , Schmitt M, Edwards GE. 1982e. *Microstegium vimineum*, a shade adapted C_4_ grass. Plant Science Letters 24: 311–318.

[CIT0111] Winter K , Wallace BJ, Stocker GC, Roksandic Z. 1983. Crassulacean acid metabolism in Australian vascular epiphytes and some related species. Oecologia 57: 129–141. doi:10.1007/BF00379570. 28310165

[CIT0112] Winter K , Medina E, Garcia V, Mayoral ML, Muniz R. 1985. Crassulacean acid metabolism in roots of a leafless orchid, *Campylocentrum tyrridion* Garay & Dunsterv. Journal of Plant Physiology 118: 73–78. 23195932 10.1016/S0176-1617(85)80166-8

[CIT0113] Winter K , Arron GP, Edwards GE. 1986a. Malate decarboxylation by mitochondria of the inducible CAM plant *Mesembryanthemum crystallinum*. Plant and Cell Physiology 27: 1533–1539.

[CIT0114] Winter K , Osmond CB, Hubick KT. 1986b. Crassulacean acid metabolism in the shade. Studies on an epiphytic fern, *Pyrrosia longifolia*, and other rainforest species from Australia. Oecologia 68: 224–230. doi:10.1007/BF00384791. 28310131

[CIT0115] Winter K , Schröppel-Meier G, Caldwell MM. 1986c. Respiratory CO_2_ as carbon source for nocturnal acid synthesis at high temperatures in three species exhibiting crassulacean acid metabolism. Plant Physiology 81: 390–394. doi:10.1104/pp.81.2.390. 16664827 PMC1075346

[CIT0116] Winter K , Lesch M, Diaz M. 1990. Changes in xanthophyll cycle components and in fluorescence yield in leaves of a crassulacean acid metabolism plant, *Clusia rosea* Jacq., throughout a 12-hour photoperiod of constant irradiance. Planta 182: 181–185. doi:10.1007/BF00197108. 24197093

[CIT0117] Winter K , Sage RF, Edwards EJ, Virgo A, Holtum JAM. 2019. Facultative crassulacean acid metabolism in a C_3_-C_4_ intermediate. Journal of Experimental Botany 70: 6571–6579. 30820551 10.1093/jxb/erz085 PMC6883265

[CIT0118] Wong SC , Hew CS. 1976. Diffusive resistance, titratable acidity, and CO_2_ fixation in two tropical epiphytic ferns. American Fern Journal 66: 121–124. doi:10.2307/1546463.

[CIT0119] Woo KC , Osmond CB. 1982. Stimulation of ammonia and 2-oxoglutarate-dependent O_2_ evolution in isolated chloroplasts by dicarboxylates and the role of the chloroplast in photorespiratory nitrogen recycling. Plant Physiology 69: 591–596. doi:10.1104/pp.69.3.591. 16662255 PMC426260

[CIT0120] Yang X , Hu R, Yin H, et al. 2017. The *Kalanchoë* genome provides insights into convergent evolution and building blocks of crassulacean acid metabolism. Nature Communications 8: 1899. 10.1038/s41467-017-01491-7 PMC5711932 29196618

[CIT0121] Zotz G , Winter K. 1996. Seasonal changes in daytime versus nighttime CO_2_ fixation of *Clusia uvitana* in situ. In: Winter K, Smith JAC. eds. Crassulacean acid metabolism. Biochemistry, ecophysiology and evolution. Berlin: Springer, 312–323.

